# Editorial: Emerging and re-emerging animal viruses: advances in diagnosis, pathogenesis, and control strategies

**DOI:** 10.3389/fcimb.2026.1919464

**Published:** 2026-08-10

**Authors:** Karam Chand, Anuj Ahuja, Shyam Singh Dahiya, Vijay Saxena

**Affiliations:** 1 ICAR- Indian Veterinary Research Institute, Nainital, Uttarakhand, India; 2 Weill Cornell Medical Center, New York-Presbyterian, New York City, NY, United States; 3 ICAR-National Institute on Foot & Mouth Disease, Bhubaneswar, Odisha, India; 4 University of Eastern Finland, Kuopio, Finland

**Keywords:** disease control, emerging and re-emerging animal viruses, genomic surveillance, host–pathogen interactions, molecular diagnostics, one health, vaccine strategies, viral diseases

Emerging and re-emerging viral diseases of animals continue to pose substantial challenges to animal health, food security, public health, and the worldwide economy. The strengthening of livestock production, climate change, altered vector habitats, wildlife–livestock–human interfaces, and international trade have accelerated the opportunities for viral emergence, cross-species transmission, and the geographic spread of pathogens. At the same time, advances in molecular biology, genomics, immunology, and surveillance have transformed our ability to identify, understand, and control these diseases. The present Research Topic *“Emerging and Re-emerging Animal Viruses: Advances in Diagnosis, Pathogenesis, and Control Strategies”* brings together a Research Topic of 13 review and research articles that highlight recent progress in viral diagnostics, epidemiological surveillance, pathogen discovery, disease pathogenesis, and prevention strategies within a One Health paradigm.

An important component of controlling emerging diseases is the development of rapid, sensitive, and field-deployable diagnostics. Early detection remains the keystone of outbreak response, particularly for diseases affecting livestock, as delays can result in extensive economic losses and increased opportunities for viruses to spread rapidly. A detailed review by Zhao et al. and Pardini et al. on one health and avian influenza provided a comprehensive overview of promising diagnostic platforms, including isothermal amplification technologies, biosensors, microfluidic systems, and avian influenza dynamics, advocating for the integration of next-generation genome sequencing with farm-level monitoring to predict and halt human spillover events.

A number of original research articles further demonstrated the translation of innovative diagnostic approaches into practical applications. Shi et al. developed a duplex crystal digital PCR assay for the differential detection of Porcine Reproductive and Respiratory Syndrome Virus, PRRSV-1 and PRRSV-2, which is one of the most economically important swine diseases worldwide. In addition, Rempel et al. evaluated the efficiency of molecular transport media for the African swine fever virus (ASFV), demonstrating that diagnostic workflows can be simplified while simultaneously enhancing biosafety during sample transportation.

Several other original research articles in this Research Topic provided important surveillance-driven studies and critical epidemiological data tracking viral lineages across new territories and unexpected hosts. Kumar et al. reported the first isolation and characterization of Japanese encephalitis virus genotype I from pigs in Assam, India. The authors documented the circulation of a genotype that has increasingly displaced genotype III in Asia and provided evidence of the virus’s presence in the nasal secretions of naturally infected pigs. This finding has implications for understanding transmission dynamics and monitoring genotype shifts relevant to vaccine effectiveness. Similarly, Narnaware et al. reported the first confirmed ASF outbreaks on the west coast of India, documenting the continued geographic expansion of ASFV genotype II. Complementing these targeted investigations, metagenomic analysis of diarrheic pig feces revealed a remarkably diverse virome, characterized by multiple co-infections and the presence of both established and potentially emerging viral pathogens. The study identified the highly lethal Genotype II lineage, underscoring the urgent need for stringent biosecurity measures as the virus continues its cross-country expansion.

Two articles in this Research Topic shifted their focus toward host-pathogen interactions and tissue-level barrier mechanisms. The mini-review by Chen et al. on porcine circovirus and autophagy synthesized the growing body of evidence that autophagic processes serve as critical interfaces between host defense mechanisms and viral replication strategies. By examining how porcine circoviruses manipulate autophagy-related pathways, the authors identified potential therapeutic targets that may help mitigate disease progression. The original research article by Schweitzer et al. investigated how neurotropic mosquito- and tick-borne orthoflaviviruses cross the Blood-Brain Barrier (BBB) model without overt disruption of barrier integrity, providing valuable clues regarding neuroinvasive mechanisms. Meanwhile, the one opinion article by Chen et al. proposed reassessment of PRRSV-induced respiratory distress, encouraging the field to move beyond traditional inflammatory paradigms and consider pulmonary surfactant dysfunction and airway obstruction as important contributors to disease pathogenesis. These perspectives stimulate new avenues of investigation that may ultimately inform therapeutic innovation.

Control and prevention strategies were another important dimension of this Research Topic. Ma et al. demonstrated the comparative analysis of E2 subunit and C-strain vaccines against classical swine fever, providing field-based evidence that supports the potential advantages of newer vaccine platforms in maintaining protective immunity and optimizing vaccination schedules. These findings demonstrate how evidence-based vaccination strategies can contribute to improved disease control while reducing the operational burden on producers. The serological investigation of foot-and-mouth disease virus exposure in dromedary camels by Rout et al. added another important viewpoint, expanding our understanding of viral ecology in understudied host species. Identifying evidence of natural exposure in camel populations raises important questions regarding their role in disease maintenance and transmission, underscoring the need for broader surveillance efforts that encompass diverse animal reservoirs.

Collectively, the articles assembled in this Research Topic illustrate the increasingly interconnected nature of animal viral diseases and the necessity of multidisciplinary approaches to address them. Advances in molecular diagnostics, genomics, surveillance, immunology, and pathogenesis research are converging to provide a more comprehensive understanding of viral emergence and control. At the same time, many challenges remain, including the need for scalable field diagnostics, enhanced surveillance networks, improved vaccine strategies, and a deeper mechanistic understanding of host–pathogen interactions.

The contributions presented here reinforce the value of a One Health perspective that recognizes the interconnectedness of animal, human, and environmental health. By integrating technological innovation with epidemiological surveillance and mechanistic research, the studies in this Research Topic advance our ability to predict, detect, and mitigate emerging and re-emerging viral threats. We hope that this Research Topic will stimulate further collaboration across disciplines and sectors, ultimately contributing to more resilient systems for animal health protection and infectious disease preparedness worldwide.

